# 

**Published:** 1883-12

**Authors:** 


### CONTENTS.

| | Page. |
|---|---|
| An Age | 239 |
| Care of the Eyes | 240 |
| Destruction of Forests | 241 |
| Croup | 242 |
| Teething | 243 |
| How to Cleanse the Waste Pipes | 245 |
| The Sick Room | 246 |
| The Evils of Hot Bread | 246 |
| Milk as Food | 247 |
| Variety in the Daily Food | 248 |
| Curative Electricity | 249 |
| A Neglected Disinfectant | 249 |
| Choose Health or Sickness | 250 |
| Memory | 250 |
| Important Decision of the Supreme Court on Imitations | 251 |
| Food Adulterations | 252 |
| Index to Volume XXX | 255, 256 |

